# Essential Oil Compounds in Combination with Conventional Antibiotics for Dermatology

**DOI:** 10.3390/molecules29061225

**Published:** 2024-03-08

**Authors:** Shivar Simbu, Ané Orchard, Sandy van Vuuren

**Affiliations:** Department of Pharmacy and Pharmacology, Therapeutic Sciences, University of the Witwatersrand, Johannesburg 2193, South Africa; 725952@students.wits.ac.za (S.S.); ane.orchard@wits.ac.za (A.O.)

**Keywords:** skin, toxicity, antimicrobial, minimum inhibitory concentration, selectivity index, synergy

## Abstract

Antimicrobial resistance has emerged as a significant threat to public health, prompting novel combinations comprising of natural sources such as essential oil compounds with conventional antibiotics. This study aimed to determine the possible interactions between six essential oil compounds with eight antibiotics/antifungals against six pathogens (*Staphylococcus aureus*, *Staphylococcus epidermidis*, *Pseudomonas aeruginosa*, *Acinetobacter baumannii*, *Cutibacterium acnes*, and *Candida albicans*) commonly implicated in skin infections. The minimum inhibitory concentrations (MICs) for the antibiotics and essential oil compounds were evaluated singularly and in combination using the broth microdilution assay. The fractional inhibitory concentrations (FIC) were calculated to determine the interactive profile of the combinations. The synergistic interactions (FIC ≤ 0.5) were further analysed at varying ratios and depicted on isobolograms. The toxicity of the synergistic combinations was determined using the brine shrimp lethality assay. Eight synergistic interactions were identified against the selected Gram-positive and *P. aeruginosa* pathogens, and the combinations also demonstrated a reduced toxicity. The combination of amoxicillin and eugenol demonstrated the lowest toxicity (LC_50_ = 1081 µg/mL) and the highest selectivity index (14.41) when in a 70:30 ratio. This study provides insight into the in vitro antimicrobial interactions of essential oil compounds and conventional antibiotics that can form a basis for newer therapies.

## 1. Introduction

Skin and soft tissue infections (SSTIs) are one of the most common types of infections in humans and occur in approximately 7–10% of all hospital patients [1,2]. These occur when there is a breakage in the epidermis layer that results in the microbial invasion of the skin or soft tissue, causing a cascade of biochemical reactions due to interactions between the host defences and pathogens [3,4]. The two main categories are uncomplicated and complicated skin infections [5]. Uncomplicated infections include superficial infections such as cellulitis or abscesses, which rarely require antibiotics and are often self-limiting [6]. In contrast, complicated infections are deep-tissue infections such as necrotizing fasciitis, which require broad-spectrum empiric antibiotic therapy or surgical intervention [5]. Although complicated infections can be treated with antibiotics, most treatment regimens are prolonged, contributing to increased antimicrobial resistance [7]. 

The injudicious use of antibiotics has allowed the dissemination of antibiotic resistance throughout the community and hospital settings, placing an ever-increasing burden on the healthcare system [8,9]. According to a recent review by Murray et al. [10], approximately 4.95 million deaths occurred in 2019 due to antimicrobial resistance. The six leading pathogens were the ESKAPE (*Enterococcus faecium, Staphylococcus aureus, Klebsiella pneumoniae, Acinetobacter baumannii, Pseudomonas aeruginosa,* and *Enterobacter)* species [11]. Moreover, these pathogens are frequently linked to skin infections, with *S. aureus* being responsible for 35–50% of all skin infections [12]. Antibiotic resistance amongst these pathogens has increased substantially, with approximately 20–100% of methicillin-resistant *S. aureus* (MRSA) clinical isolates showing high resistance to several antibiotic classes, including glycopeptides [10,13]. Gram-negative pathogens such as *A. baumannii* have also exhibited widespread resistance, with approximately 90% of clinical isolates being resistant to meropenem [14,15,16], whilst *P. aeruginosa’s* resistance rates have increased by between 15 to 30%, with some isolates showing extensively drug-resistant (XDR) profiles [17]. Antifungal resistance has also emerged as another public health crisis [18,19]. Recent epidemiological data suggest that fungal infections contribute to approximately 13 million infections annually with roughly 1.5 million deaths worldwide [20,21]. Candidiasis is the most common fungal infection, with *C. albicans* accounting for 70–90% of all infections [18]. The continuous increase in antimicrobial resistance in contrast to the slow development of new antimicrobial agents has expedited the need for alternative approaches, such as the investigation of natural antimicrobial agents from essential oils and related compounds. 

Essential oils (EOs) are complex natural mixtures of bio-active compounds produced by plants as secondary metabolites and have been used for centuries to treat various diseases and ailments [22,23]. An EO is composed of between 20 and >200 different chemical compounds with varying molecular structures and can be broadly classified into three groups: terpenes, terpenoids, and non-terpene-derived compounds called phenylpropanoids [24,25]. These essential oil compounds (EOCs) are responsible for the pharmacological properties of an EO and, more specifically, their antimicrobial properties [24]. EOCs have been studied independently and are actively being sought as novel chemical entities for antimicrobial development [25,26].

In the past decade, antimicrobial combination therapy has become the mainstay for many clinically problematic infections requiring higher-order combinations, especially for nosocomial infections [11,17]. Many authors have argued and even alluded to the hypothesis that EOCs could be combined with conventional antibiotics as a type of syncretic combination [27,28,29]. Numerous studies have documented in vitro interactions of combinations between EOCs and conventional antibiotics with varying outcomes, which have been reviewed extensively [22,30,31,32]. However, there are limited studies investigating combinations against skin pathogens that have elucidated the type of interactions that can occur between the EOCs and any conventional antibiotic at varying ratios [33]. Since EOCs elicit broad-spectrum antimicrobial activity via multitarget mechanisms, combinations with conventional antibiotics could be a novel approach to reduce or attenuate resistance in pathogens, allowing ineffective antibiotics to be reclaimed [23]. 

Despite the overwhelming evidence which supports the antimicrobial properties of EOCs, the toxicity of these compounds remains understudied and has been recommended for further investigation [34]. Many EOCs elicit a high inherent toxicity due to their lipophilic nature, which causes damage to cell membranes and induces oxidative stress [35]. Several reviews have discussed various methods, such as formulating EOCs into nano-emulsions or adding adjunctive agents which can reduce the toxicity of individual EOCs [36,37], with only a handful of studies investigating the overall toxicity of these combinations [38,39].

This study evaluated the effects of combining essential oil compounds with conventional antibiotics against common skin pathogens to elucidate the effectiveness of these combinations with regard to their overall antimicrobial effect and toxicity.

## 2. Results and Discussion

### 2.1. Antimicrobial Analysis 

The MIC results for conventional antimicrobials and essential oil compounds are presented in Table 1. For the conventional antibiotics, Gram-positive bacteria showed a greater susceptibility than the Gram-negative bacteria, which is a common observation due to physiological differences between the two types of bacteria [11]. *Cutibacterium acnes* showed the greatest susceptibility to conventional antibiotics, particularly erythromycin (0.12 µg/mL) and meropenem (0.20 µg/mL). All the Gram-positive bacteria demonstrated sensitivity to miconazole, with MIC values ranging from 0.63 to 1.88 µg/mL, which is in agreement with several previous studies [40,41]. Miconazole is an imidazole typically used for superficial cutaneous fungal infections [42]. Unlike other azole antifungal drugs, miconazole elicits antibacterial activity that may be effective in polymicrobial infections [43]. In contrast, the Gram-negative bacteria showed no susceptibility to miconazole, with some studies demonstrating MIC values > 1000 µg/mL [41,43]. For *C. albicans*, miconazole (0.78 µg/mL) showed better activity when compared to nystatin (1.56 µg/mL), which had also been demonstrated in previous studies [44]. 

All the EOCs showed varying degrees of antimicrobial activity against all the reference strains, with cinnamaldehyde (MIC 458 µg/mL) being the most effective. These results agree with previous studies that have discussed that the variability in an EOC’s antimicrobial activity is primarily based on the chemical nature, chirality, and hydrophobic/hydrophilic nature of the compound [27,45,46]. Cinnamaldehyde (458 µg/mL) demonstrated better antimicrobial effects compared to eugenol, which differs from previous studies [47], with the consensus being that eugenol is more effective than cinnamaldehyde and exhibits a higher activity against Gram-negative bacteria [48]. These observations may imply that certain EOCs are better suited for a specific type of micro-organism. Both α-pinene and γ-terpinene showed a higher antimicrobial activity against *C. albicans* than the bacteria, which had also been demonstrated by previous studies [49]. However, Silva et al. [50] showed that α-pinene had no activity against *C. albicans* and suggested that racemic mixtures of both enantiomers would be more effective. Both the negative and culture control did not affect the growth of the bacterial and fungal cultures. 

### 2.2. Combinations 

The results for the 1:1 combinations against the Gram-positive bacteria are presented in Table 2. A total of 126 combinations were studied with seven (5%) being synergistic, 68 (54%) being additive, and 51 (40%) demonstrating non-interactive interactions. No antagonistic interactions were observed. Four synergistic interactions were observed against *S. aureus*: amoxicillin with carvacrol (ΣFIC 0.42), gentamicin with carvacrol (ΣFIC 0.36), miconazole with carvacrol (ΣFIC 0.33), and miconazole with cinnamaldehyde (ΣFIC 0.32). Two synergistic interactions were noted against *S. epidermidis*: erythromycin with cinnamaldehyde (ΣFIC 0.44) and miconazole with cinnamaldehyde (ΣFIC 0.07). For *C. acnes*, combining amoxicillin with eugenol resulted in synergy (ΣFIC 0.30). The synergistic interactions highlight the potential of carvacrol and cinnamaldehyde against Gram-positive bacteria. Previous studies had elucidated similar results involving carvacrol and cinnamaldehyde against *S. aureus* and *S. epidermidis* [51,52]. However, in contrast, there are limited studies investigating *C. acnes*, which may be attributed to its low virulence potential [53]. The investigation of miconazole’s anti-staphylococcal properties is often overlooked in the literature, with no previous studies investigating the interactive profiles of miconazole and EOCs against Gram-positive bacteria. From the results observed, it was noted that several interactions were additive (28%), demonstrating some feasibility for inclusion. Several additive interactions were noted for combinations involving meropenem against all Gram-positive bacteria studied, possibly due to meropenem’s strong antibacterial activity and high barrier to resistance [54].

**Figure 1:**
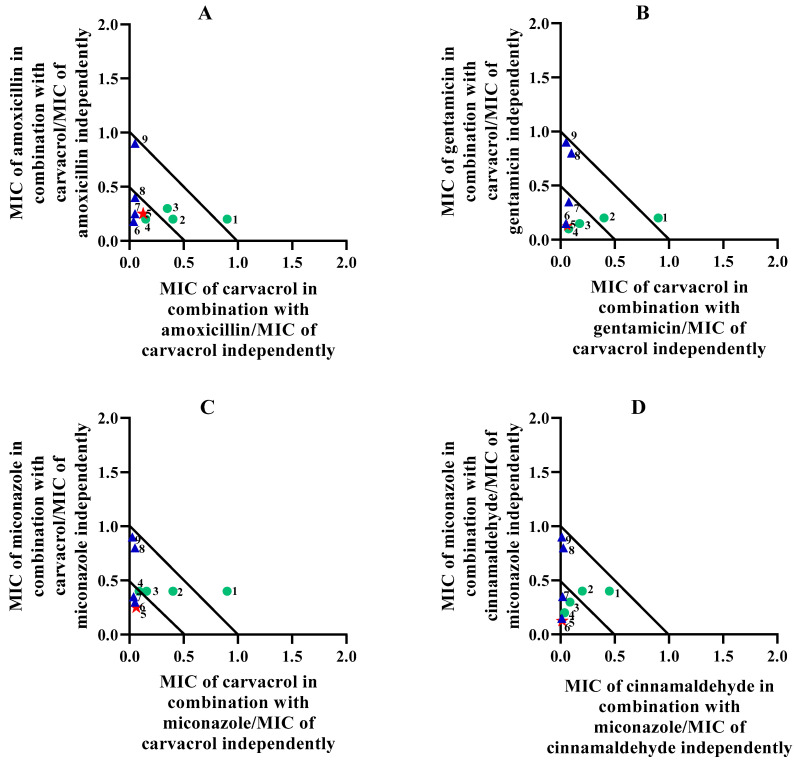

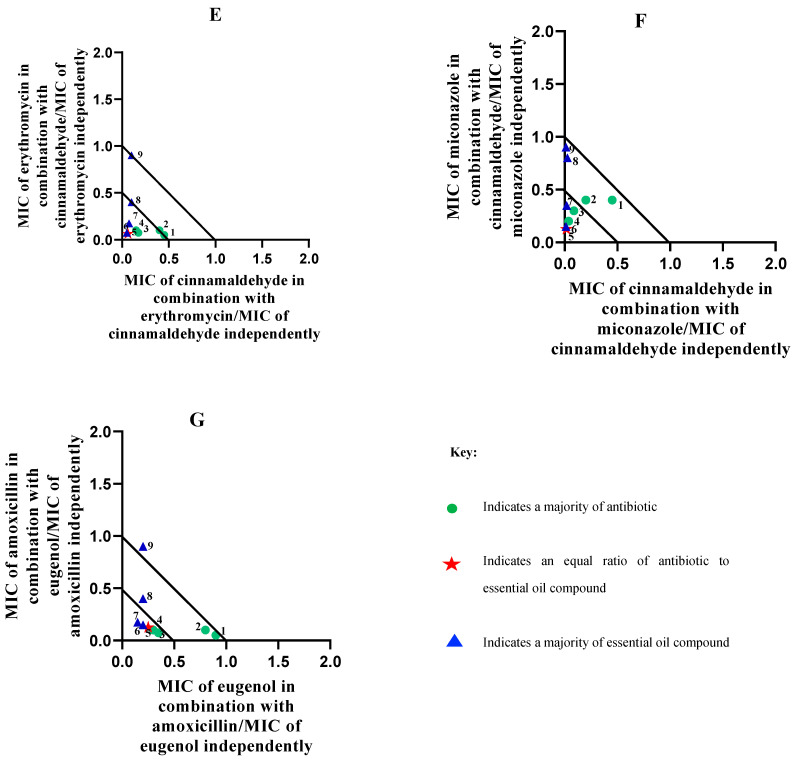
(**A**–**G**): Isobologram representation of synergistic interactions between different antibiotics and EOCs against Gram-positive bacteria. (**A**–**D**) was against *S. aureus* (ATCC 25923); (**E**,**F**) was against *S. epidermidis* (ATCC 12228); and (**G**) was against *C. acnes* (ATCC 11827).

The synergistic interactions were further analysed in varied ratio concentrations and presented on isobolograms (Figure 1A–G). For *S. aureus*, three synergistic interactions were observed involving carvacrol with three antibiotics (Figure 1A–C). Comparatively, these combinations showed a similar trend in the ratio of antibiotic to EOC, with ratios of 60:40, 50:50, and 40:60 showing the highest degree of synergy. Figure 1D shows the isobologram of miconazole and cinnamaldehyde against *S. aureus,* which demonstrated higher levels of synergy (lower ΣFIC values) compared to the isobologram depicting the combination of miconazole and carvacrol (Figure 1C) against *S. aureus*. Figure 1E and F represent the isobolograms of synergistic combinations against *S. epidermidis*. The combination of erythromycin with cinnamaldehyde demonstrated the highest synergy compared to the combination of miconazole with cinnamaldehyde. In addition, six out of the nine ratios for the combination of erythromycin with cinnamaldehyde were synergistic. The combination of miconazole with cinnamaldehyde was synergistic against both *S. aureus* (Figure 1D) and *S. epidermidis* (Figure 1F). Very similar results in terms of synergistic ratios were noted, which highlights the potential of this combination. The isobologram for *C. acnes* is presented in Figure 1G. The general trend for *C. acnes* was that greater degrees of synergy were achieved when the ratio of antibiotic to EOC approached equal parts (1:1). In comparison to the combination of amoxicillin with carvacrol against *S. aureus* (Figure 1A), a similar trend was observed particularly at ratios where the EOC made up the majority of the concentration (10:90). 

Several reviews have discussed carvacrol and cinnamaldehyde against food-borne pathogens [55,56], with the general consensus being that carvacrol has more potent antibacterial effects on Gram-positive bacteria whilst cinnamaldehyde has a more broad-spectrum effect against bacteria and fungi [57]. In the context of combinations, cell membrane damage brought about by carvacrol or cinnamaldehyde may increase the intracellular concentration of an antibiotic due to allosteric modulatory mechanisms that results in reduced MICs [46,48]. However, further studies are needed to investigate these mechanisms. The synergistic interactions elucidated in this study between miconazole, carvacrol, and cinnamaldehyde against *S. aureus* and *S. epidermidis* highlight its potential as a novel candidate broad-spectrum topical agent. Considering that most skin lesions are perpetuated by the presence of bacteria which may also increase the virulence of some fungal species [58], miconazole’s combined antifungal and antibacterial properties may be an effective treatment. This has already been demonstrated in mixed fungal–bacterial infections such as dermatomycoses, with some studies suggesting miconazole’s direct involvement in reducing the severity of this type of infection [59]. 

The results for the 1:1 combinations against Gram-negative bacteria are presented in Table 3. A total of 48 combinations, which only included the conventional antibiotics that had activity against Gram-negative bacteria. From the results, 40% (19/48) of the combinations were additive, while the remaining 58% (28/48) were non-interactive.

**Figure 2:**
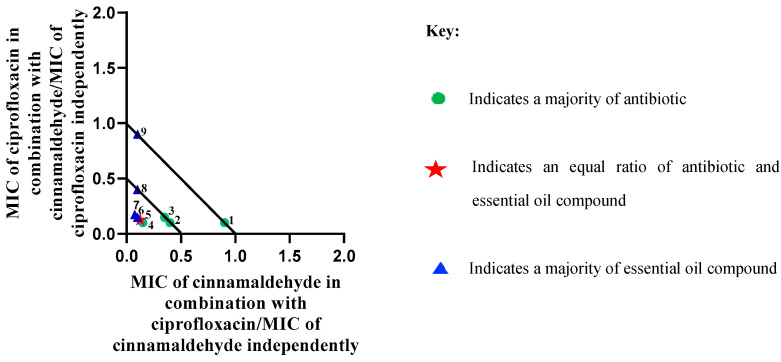
Isobologram represents synergistic interactions between ciprofloxacin and cinnamaldehyde against *P. aeruginosa* (ATCC 27853).

One synergistic interaction (ΣFIC 0.32) was observed against *P. aeruginosa*. Most combinations (16/24) against *P. aeruginosa* were additive, with combinations involving ciprofloxacin showing more favourable results than the rest. Figure 2 represents varied ratios for the synergistic interaction of ciprofloxacin with cinnamaldehyde against *P. aeruginosa*. In addition to the ratios in which the EOC and antibiotic components were in equal parts, the ratios (antibiotic/EOC) of 60:40, 40:60, and 30:70 also demonstrated synergy. 

Due to its high virulence potential and intrinsic resistance to several antibiotic classes [11,60], many studies have attempted to find combinations of EOCs and antibiotics against *P. aeruginosa*. However, only some have elucidated the precise synergistic combinations, with the majority being non-interactive [46]. A study by Miladinović et al. [61] demonstrated that geraniol and thymol were synergistic with tetracycline and chloramphenicol against *P. aeruginosa.* Recent studies [62,63] demonstrated synergistic interactions (ΣFIC 0.37–0.50) involving gentamicin and colistin in combination with cinnamaldehyde against the PA01 strain of *P. aeruginosa*. These antibiotics exhibit poor permeability through the pseudomonal outer membrane as well as being extruded out of the cell by efflux pumps [60]. From the results, the majority (17/24) of the combinations against *A. baumannii* were non-interactive (ΣFIC 1.07–2.20). However, gentamicin with eugenol (ΣFIC 0.62) and meropenem with eugenol (ΣFIC 0.64) demonstrated additivity. 

Previous studies investigating *A. baumannii* have used essential oils such as *Coriandrum sativum* L. (coriander) and *Origanum vulgare* L. (oregano) with conventional antibiotics, with some studies reporting that certain essential oils can increase the sensitivity of *A. baumannii,* resulting in synergy [64,65]. However, there are limited studies investigating EOCs against *A. baumannii* [66]. Karumathil et al. [67] demonstrated that triple combinations of cinnamaldehyde and eugenol with some beta-lactams enhanced the sensitivity of *A. baumannii* to these antibiotics, resulting in lower MIC values and reduced bacterial cell counts. Aleksic Sabo et al. [68] reported that combinations of carvacrol, thymol, and eugenol with ciprofloxacin were synergistic (ΣFIC 0.11–0.50) against both reference and multi-drug resistant strains of *A. baumannii,* which differs from the results in this study, in which non-interactive interactions were observed (ΣFIC 1.27–1.60). A possible reason for this difference may be due to the solvent used in the MIC assays or the overall resistance profiles of the *A. baumannii* strains. There are limited studies investigating interactions between EOCs and conventional antibiotics against Gram-negative bacteria and fungi. Much of the focus has shifted to investigating how EOCs can attenuate virulence factors and resistance mechanisms [23,25,26]. Based on current therapies, many existing antibiotic strategies rely on combinations of an antibiotic with an adjuvant to target a resistance mechanism and “resensitize” the micro-organism to the antibiotic [16]. 

A well-known example of this is coupling beta-lactams with beta-lactamase inhibitors [69]. This strategy is often very effective and EOCs in combination with conventional antibiotics may be the best way to approach this type of combination therapy. Many studies published in the last five years have investigated the ability of certain EOCs to attenuate resistance mechanisms such as efflux pumps and beta-lactamase enzymes [67,70,71,72,73]. These studies have shown that EOCs can downregulate and even inactivate specific resistance mechanisms, re-establishing the antibiotic’s effectiveness against the pathogen. Therefore, the results from this study support the concept that EOCs can be used as adjunctive agents alongside conventional antibiotics. 

For *C. albicans*, 75% of the combinations (Table 4) were non-interactive (ΣFIC 1.02–2.13), whilst 25% were additive (ΣFIC 0.77–0.90). Eugenol showed the lowest overall ΣFIC values when combined with miconazole (0.87) and nystatin (0.77), whilst α-pinene had the highest ΣFIC values of 2.13 (miconazole) and 1.73 (nystatin). Eugenol exhibited noteworthy antifungal activity against various fungal species due to its ability to damage the fungal cell envelope and attenuate virulence factors [74]. Several studies have demonstrated synergistic interactions between eugenol and various antifungal agents, including fluconazole, micafungin, and amphotericin B, against *C. albicans* and related species [75,76,77,78,79]. These studies highlight the effectiveness of eugenol in combination with conventional antifungals against fungi. In this study, most of the interactions against *C. albicans* were non-interactive, which could be based on the fact that both the antifungal agent and the EOC may compete for the same molecular target [80]. Both nystatin and miconazole interact differently with the fungal cell membrane, which is also the primary molecular target for most EOCs [20,74]. Considering that EOCs can have multiple mechanisms of action which act sequentially and are not selective for a specific target site [48,80,81], the EOC may compete with the antifungal agent for the binding site and reduce the overall effectiveness of the combination.

### 2.3. Toxicity Analysis

The toxicity of the conventional antimicrobials is presented in Table 5. Based on the results, none of the conventional antimicrobials were considered toxic (LC_50_ > 1000 µg/mL), which can be attributed to the selectivity of the antibiotics for prokaryotes as opposed to eukaryotes [82]. In addition, the negative controls did not affect the outcome of the assay. 

The toxicity values of the EOCs expressed as LC_50_ after 24 h and 48 h of exposure are presented in Table 6. From the results for the 24 h of exposure, both α-pinene and γ-terpinene were considered non-toxic (LC_50_ > 1000 µg/mL), with the remaining four compounds (linalool, eugenol, carvacrol, and cinnamaldehyde) showing high toxicity (LC_50_ = 64.43–77.62 µg/mL). All EOCs showed a decrease in the LC_50_ after 48 h, except for linalool, which showed an increase (LC_50_ = 84.12 µg/mL) in toxicity, which may have been due to the pharmacokinetics and chemical stability of the compound [83].

Carvacrol showed the highest increase in toxicity (LC_50_ = 37.14 µg/mL), with both α-pinene and γ-terpinene remaining non-toxic after 48 h. Previous studies have investigated the toxic effects of EOCs, with varying results based on the studied model [85,86]. Pattanasiri et al. [87] reported eugenol to cause 100.00% mortality in Siamese fighting fish at a concentration of 0.04 mg/mL, which supports the findings of this study, as 100.00% mortality of brine shrimp was observed at doses of 0.50 and 1.00 mg/mL. Youssefi et al. [88] demonstrated that carvacrol elicited a high toxicity in mosquito larvae with an LC_50_ = 15 µg/mL. Terpenoids such as eugenol and linalool are known for their high toxicity, whilst lower LC_50_ values are typically recorded for terpenes such as α-pinene and γ-terpinene [89]. 

The toxicity of the antimicrobial synergistic combinations is presented in Table 7. All the combinations showed weak/low toxicity (LC_50_ = 500–999 µg/mL) with the combination of ciprofloxacin and cinnamaldehyde being the least toxic (LC_50_ = 827.92 µg/mL) after 24 h, and the combination of gentamicin and carvacrol being the least toxic (LC_50_ = 696.07 µg/mL) after 48 h. A comparison of the toxicity of individual EOCs alone and when combined with conventional antibiotics shows a notable decrease in toxicity. This suggests that the conventional antibiotics used attenuate the toxicity of the EOCs. To the best of our knowledge, there have been minimal studies investigating the toxicity of combinations comprising conventional antibiotics and EOCs, supporting the novelty of this study.

### 2.4. Selectivity Index 

The LC_50_ and SI for each synergistic ratio are presented in Table 8. Most of the ratios demonstrated a low toxicity (LC_50_ = 500–999 µg/mL), with the general trend being that increasing the amount of the EOC increased the toxicity. The combination of gentamicin and carvacrol at a ratio of 70:30, with the antibiotic making up the majority of the compound, was non-toxic, with an LC_50_ = 1025.32 µg/mL after 24 h. It showed a weak/low toxicity (LC_50_ = 931.44 µg/mL) after 48 h. In addition, the combination of amoxicillin and eugenol at a ratio of 70:30, with the antibiotic making up the majority of the compound, was also non-toxic, with an LC_50_ = 1081.17 µg/mL after 24 h and an LC_50_ = 843.04 µg/mL after 48 h.

For the SI values, the combination of amoxicillin and eugenol at a ratio of 70:30 demonstrated the highest SI of 14.41 at 24 h and 11.23 at 48 h. The lowest SI was noted for combinations comprising amoxicillin and carvacrol ranging from 0.31 to 1.05. Based on the results from Table 8, several combinations warrant further investigation, particularly against cell lines or a comparative model. 

## 3. Materials and Methods 

The overall experimental design can be found in Figure S1 (Supplementary Materials). 

### 3.1. Preparation of Cultures

Due to their prevalence in skin infections, the following six American Type Culture Collection (ATCC) reference strains were included in this study: *Staphylococcus aureus* (ATCC 25923), *Staphylococcus epidermidis* (ATCC 12228), *Acinetobacter baumannii* (ATCC 19606), *Pseudomonas aeruginosa* (ATCC 27853), *Cutibacterium acnes* (ATCC 11827), and *Candida albicans* (ATCC 10231). All the bacteria were cultured in Tryptone Soya broth (TSB) (Oxoid) and incubated at 37 °C for 24 h, except for *C. acnes*, which was inoculated into Thioglycolate broth (TGB) (Oxoid) and incubated for seven days under anaerobic conditions (5.00% CO_2_) at 37 °C. *Candida albicans* was cultured in TSB and incubated at 37 °C for 48 h. In addition, streak plates were prepared to ensure purity. 

### 3.2. Antimicrobial Agents and Essential Oil Compounds 

Based on their use in skin infections, the following antibiotics/antifungals (Sigma-Aldrich, St. Louis, MO, USA) were included; amoxicillin (potency ≥ 90.0%), ciprofloxacin (≥98.0%), erythromycin (≥85.0%), tetracycline (≥98.0%), gentamicin (≤100%), meropenem (≥95.0%), miconazole (99.5%), and nystatin (≥95.0%). The antibiotics were only tested against the micro-organisms in which direct antibacterial activity had been noted. Miconazole was also tested against Gram-positive bacteria due to its known antibacterial activity [41,90]. The antibiotic/antifungal stocks were prepared as outlined by the Clinical and Laboratory Standards Institute (CLSI) [91]. Stock solutions (0.01 mg/mL and 0.05 mg/mL) were stored at −20 °C, and working stocks were stored at 4 °C. 

Based on the previous literature which has documented EOCs’ antimicrobial properties [28,33,51], the following EOCs (Sigma-Aldrich, St. Louis, MO, USA) were selected: α-pinene, γ-terpinene, ±Linalool, eugenol, carvacrol, and cinnamaldehyde. For the combinations, the compounds were only combined with antibiotics/antifungals that had demonstrated direct antimicrobial activity against the tested organism. All the compounds had a purity range of 95.00–99.00%. The compounds were stored at 4 °C and prepared to a starting concentration of 32.00 mg/mL. 

### 3.3. Minimum Inhibitory Concentration (MIC) 

The broth microdilution assay (MIC) was used to evaluate the antimicrobial activity of the conventional antimicrobials and the selected EOCs independently and in combination [92]. Briefly, each well of a 96-well microtiter plate was filled with 100 µL of their respective sterile broth, followed by adding 100 µL of the antibiotic/antifungal or EOC into the top row. For the combinations, 50 µL of each antibiotic/antifungal and 50 µL of each EOC were added so that the ratio of antibiotic/antifungal to the EOC was 1:1. Serial doubling dilutions were then performed. The prepared microtiter plates were then inoculated with 100 µL of the relevant pathogen at colony-forming units (CFU) of approximately 1 × 10^6^ (CFU/mL). The plates were then sealed with sterile adhesive to ensure no sample loss since EOCs are volatile. The plates were then incubated at their respective temperature and times. After incubation, 40.00 µL of a 0.40% *p*-iodonitrotetrazolium (INT) violet indicator solution was added to all the inoculated wells. A change in the colour of the wells from clear to pink or red was used to indicate the presence of microbial growth [93]. The MIC values were interpreted as the lowest concentration at which growth was inhibited. Each combination was performed in quadruplicate. Three controls were included: a culture control for the broth corresponding to the sample, a solvent control, and a conventional antimicrobial control to ensure susceptibility. The mean and standard deviations (± SD) for the MICs were calculated in MS Office (2016). 

### 3.4. Interactive Profiles 

The interactions between the combinations of the antibiotics and compounds were classified according to their fractional inhibitory concentration (FIC) (Equation (1)).

(1) $$\mathrm{FIC}\;(I)=\frac{\;\left(A\right)\;\mathrm{combined}\;\mathrm{with}\;(B)}{\left(A\right)\;\mathrm{independently}\;}\;\mathrm{FIC}\;(\mathrm{II})=\frac{\;\left(B\right)\;\mathrm{combined}\;\mathrm{with}\;(A)}{\;\left(B\right)\;\mathrm{independently}\;}$$

where (A) is the MIC of the essential oil compound, and (B) is the MIC of the antibiotic.

From these values, the ΣFIC was calculated following Equation (2):

ΣFIC = FIC (I) + FIC (II)(2)

The ΣFIC for each EO compound combined with an antibiotic was interpreted as follows; an ΣFIC value of ≤0.5 was indicative of synergy; an ΣFIC value of >0.5–1.0 indicated an additive interaction; an ΣFIC of >1.0–≤ 4.0 indicated non-interactive; and an ΣFIC value of >4.0 indicated antagonism [94]. 

### 3.5. Varied Ratio Combinations 

For notable combinations demonstrating synergistic interactions in the 1:1 ΣFIC analysis, a further study was undertaken during which combinations were investigated at varied ratios of 90:10, 80:20, 70:30, 60:40, 50:50, 40:60, 30:70, 20:80, and 10:90. The data points for each ratio were then plotted on an isobologram using the GraphPad Prism^®^ software (Version 9). Synergy was displayed for the data points closest to the apex and falling beneath or on the 0.5:0.5 line. Additive interactions referred to the data points between the 0.5:0.5 and the 1:1 lines. Non-interactive effects were those that were between the 1:1 and the 4:4 lines and antagonism was displayed for the data points above or on the 4:4 line [94].

### 3.6. Toxicity Studies 

The brine shrimp lethality assay was used to determine the toxicity of the EOCs, antibiotics, and combinations demonstrating synergy [95]. An amount of 0.50 g of dried brine shrimp (*Artemia franciscana*) eggs (Ocean Nutrition) was weighed out and added to artificial seawater (16.00 g of sea salt (TropicMarine) in 500.00 mL of distilled water). A rotary pump was used to aerate the sea water to increase the hatch rate. The eggs were left for 48 h under a light source to allow the brine shrimp to hatch. A 48-well microtiter plate was prepared for the assay by adding 400.00 µL of saltwater containing 40–60 live brine shrimp to each well. A volume of 400.00 µL of either the EOC or antibiotic was added to each well. For the synergistic combinations, 200.00 µL of each sample (antibiotic/EOC) was mixed prior to adding to the wells. Before adding the samples, each well was examined using a light microscope (Olympus, 40× magnification) to check the viability of the brine shrimp. Six concentrations (2.0, 1.0, 0.5, 0.25, 0.125, and 0.06125 mg/mL) of each EOC were prepared using 2.00% DMSO and diluted in the well to achieve a 1:2 final concentration. All the antibiotic samples were prepared to a 0.1 mg/mL concentration using sterile distilled water. At 24 h and 48 h, the dead shrimp were viewed and counted. A lethal dose of 50 µL acetic acid (Saarchem) was added to each well. Thereafter, the percentage of mortality was calculated, and a percentage of 50% mortality or greater was considered biologically toxic [84]. The assay included a negative, non-toxic control of 32.00 g/L of artificial sea water to ensure the promotion of growth and the survival of the brine shrimp. The positive (toxic) control in the assay consisted of a 1.60 mg/mL potassium dichromate solution. The brine shrimp mortality was plotted against the logarithms of the concentrations using the Probit analysis tool in the IBM SPSS Statistics software (Version 27). The median lethal concentration (LC_50_) at 95% confidence intervals (CI) was calculated [96]. 

### 3.7. Selectivity Index (SI) 

The SI can be defined as the ratio of the toxic concentration of a sample against its effective bio-active concentration [36]. The SI represents the pre-clinical screening calculation used to determine the feasibility of novel compounds for in vivo testing [82]. When evaluating the SI, a cut-off value of ≥4 was used [97,98]. The SI was calculated using Equation (3) for each of the synergistic combinations.

(3) $$\mathrm{SI}=\frac{{\mathrm{LC}}_{50}*}{\mathrm{MIC}}$$

* where the LC_50_ is the lethal concentration required to cause 50% mortality after 24 h and 48 h, and the MIC is the minimum inhibitory concentration of the antimicrobial synergistic combination. 

## 4. Conclusions

This study investigated the emerging strategy of combining essential oil compounds with conventional antibiotics against six reference skin pathogens. Based on the findings, 48.39% of combinations were additive, 47.31% were non-interactive, and 4.30% were synergistic, with no antagonism observed. In addition, eight synergistic interactions were identified, seven being effective against Gram-positive bacteria and one against *P. aeruginosa*. Two synergistic interactions involving miconazole with two different EOCs were identified against *S. aureus* and *S. epidermidis,* which was the first to be reported. Furthermore, this study showed that synergistic interactions can exist at varying ratios for combinations of EOCs and conventional antibiotics. For *C. albicans,* it may be worth investigating combinations of different antifungals and EOCs at varying ratios to elucidate the potency of eugenol at different concentrations. Although the individual EOCs have high inherent toxicities, the overall toxicity can be reduced when combined with conventional antimicrobials. In addition, the combination of amoxicillin and eugenol demonstrated an SI of 14.41, which warrants further investigation. Therefore, it can be proposed that some essential oil compounds enhance the antimicrobial efficacy of some conventional antibiotics. 

## Figures and Tables

**Table 1:** The mean MIC values (µg/mL) and standard deviation (±SD) of conventional antimicrobials and essential oil compounds against the pathogens (*n* = 6).

Conventional Antibiotic	Micro-Organisms					
	*S. aureus* (ATCC 25923)	*S. epidermidis* (ATCC 12228)	*C. acnes* (ATCC 11827)	*P. aeruginosa* (ATCC 27853)	*A. baumannii* (ATCC 19606)	*C. albicans* (ATCC 10231)
Amoxicillin	0.90 ± 0.13	0.51 ± 0.16	0.24 ± 0.09	NS ^1^	NS	NS
Ciprofloxacin	1.06 ± 0.24	0.94 ± 0.36	1.25 ± 0.00	0.57 ± 0.28	0.52 ± 0.16	NS
Erythromycin	0.63 ± 0.00	0.42 ± 0.22	0.12 ± 0.06	NS	NS	NS
Gentamicin	1.41 ± 0.18	3.13 ± 0.00	1.56 ± 0.00	0.78 ± 0.00	2.73 ± 0.78	NS
Meropenem	3.13 ± 0.00	3.52 ± 0.45	0.20 ± 0.00	0.78 ± 0.00	1.53 ± 0.31	NS
Tetracycline	1.25 ± 0.00	1.25 ± 0.00	0.70 ± 0.09	18.75 ± 7.21	3.91 ± 1.91	NS
Miconazole	1.88 ± 0.96	1.72 ± 0.64	0.63 ± 0.00	NS	NS	0.78 ± 0.29
Nystatin	NS	NS	NS	NS	NS	1.56 ± 0.63
**Essential Oil Compounds**						
α-Pinene	6000 ± 0.00	4000 ± 0.00	1500 ± 577	3750 ± 250	4000 ± 0.00	1500 ± 577
γ-Terpinene	4000 ± 0.00	3000 ± 1154	1500 ± 577	3500 ± 1788	4000 ± 0.00	2500 ± 1000
±Linalool	2750 ± 1035	4000 ± 0.00	1500 ± 547	3000 ± 1673	2000 ± 0.00	1250 ± 500
Eugenol	1667 ± 517	2000 ± 0.00	1000 ± 0.00	1500 ± 837	1500 ± 837	750 ± 288
Carvacrol	2000 ± 0.00	750 ± 274	500 ± 0.00	750 ± 478	1000 ± 0.00	500 ± 0.00
Cinnamaldehyde	417 ± 129	1000 ± 250	208 ± 65.0	500 ± 0.00	500 ± 0.00	125 ± 0.00
Negative control	>8000 ± 0.00	>8000 ± 0.00	>8000 ± 0.00	>8000 ± 0.00	>8000 ± 0.00	>8000 ± 0.00
Culture control	>8000 ± 0.00	>8000 ± 0.00	>8000 ± 0.00	>8000 ± 0.00	>8000 ± 0.00	>8000 ± 0.00

^1^ NS—not susceptible.

**Table 2:** The FIC values for combinations of conventional antibiotics (A) and EOCs (B) against Gram-positive bacteria (*n* = 4).

Conventional Antibiotic	Essential Oil Compounds	*S. aureus* (ATCC 25923)			*S. epidermidis* (ATCC 12228)			*C. acnes* (ATCC 11827)		
		FIC(A) ^1^	FIC(B) ^2^	ΣFIC ^3^	FIC(A)	FIC(B)	ΣFIC	FIC(A)	FIC(B)	ΣFIC
**Amoxicillin**	α-pinene	0.69	0.33	1.02	0.62	0.25	*0.87*	0.50	0.25	*0.75*
	γ-terpinene	0.69	0.50	1.19	0.92	0.50	1.12	0.67	0.33	*1.00*
	±Linalool	0.69	0.73	1.42	0.62	0.25	*0.87*	0.67	0.33	*1.00*
	Eugenol	0.35	0.60	*0.85*	0.31	0.25	*0.56*	0.17	0.13	**0.30**
	Carvacrol	0.17	0.25	**0.42**	0.31	0.67	*0.98*	0.33	0.50	*0.83*
	Cinnamaldehyde	0.17	1.20	1.37	0.31	0.50	*0.81*	0.25	0.90	1.15
**Ciprofloxacin**	α-pinene	0.59	0.33	*0.92*	0.67	0.50	1.17	0.50	1.33	1.85
	γ-terpinene	0.29	0.25	*0.54*	0.33	0.33	*0.66*	0.50	1.33	1.83
	±Linalool	0.59	0.73	1.32	0.33	0.25	*0.58*	0.50	1.33	1.83
	Eugenol	0.59	1.20	1.79	0.50	0.75	1.25	0.25	1.00	1.25
	Carvacrol	0.29	0.50	*0.79*	0.17	0.67	*0.84*	0.13	1.00	1.13
	Cinnamaldehyde	0.15	1.20	1.35	0.17	0.50	*0.67*	0.06	1.20	1.26
**Erythromycin**	α-pinene	0.50	0.17	*0.67*	2.99	1.00	3.99	0.50	0.13	*0.63*
	γ-terpinene	0.50	0.25	*0.75*	1.50	0.67	2.17	0.67	0.17	*0.84*
	±Linalool	0.50	0.37	*0.87*	1.50	0.50	2.00	0.67	0.17	*0.84*
	Eugenol	0.50	0.60	1.10	1.12	0.75	1.87	1.00	0.38	1.38
	Carvacrol	0.50	0.50	*1.00*	0.75	1.33	2.08	0.67	0.50	1.17
	Cinnamaldehyde	0.13	0.60	*0.73*	0.19	0.25	**0.44**	0.67	1.20	1.87
**Gentamicin**	α-pinene	0.44	0.33	*0.77*	0.50	0.83	1.33	1.00	0.67	1.67
	γ-terpinene	0.44	0.50	*0.94*	0.50	2.00	2.50	1.00	0.67	1.67
	±Linalool	0.44	0.73	1.17	0.50	0.25	*0.75*	1.00	0.67	1.67
	Eugenol	0.22	0.60	*0.82*	0.50	0.50	*1.00*	0.50	0.50	*1.00*
	Carvacrol	0.11	0.25	**0.36**	0.25	0.67	*0.92*	0.25	0.50	*0.75*
	Cinnamaldehyde	0.08	0.60	*0.68*	0.25	0.50	*0.75*	0.12	0.60	*0.72*
**Meropenem**	α-pinene	0.50	0.17	*0.67*	0.44	0.25	*0.69*	0.50	0.04	*0.54*
	γ-terpinene	0.50	0.25	*0.75*	0.44	0.33	*0.77*	1.00	0.08	1.08
	±Linalool	0.50	0.36	*0.86*	0.44	0.25	*0.69*	0.50	0.04	*0.54*
	Eugenol	0.25	1.20	1.45	0.44	0.50	*0.94*	0.50	0.06	*0.56*
	Carvacrol	0.38	0.38	*0.76*	0.22	0.67	*0.89*	0.50	0.12	*0.62*
	Cinnamaldehyde	0.25	1.20	1.45	0.22	0.50	*0.72*	0.50	0.30	*0.80*
**Miconazole**	α-pinene	0.67	0.67	1.34	0.36	0.50	*0.86*	1.00	1.33	2.33
	γ-terpinene	0.33	0.50	*0.83*	0.36	0.67	1.03	1.00	1.33	2.33
	±Linalool	0.33	1.46	1.79	0.36	0.50	*0.86*	0.50	0.67	1.17
	Eugenol	0.33	1.20	1.53	0.36	1.00	1.36	1.25	0.50	1.75
	Carvacrol	0.08	0.25	**0.33**	0.14	1.00	1.14	0.12	0.50	*0.62*
	Cinnamaldehyde	0.02	0.30	**0.32**	0.01	0.06	**0.07**	0.06	0.60	*0.66*
**Tetracycline**	α-pinene	0.50	0.33	*0.83*	0.50	0.50	*1.00*	0.89	1.33	2.20
	γ-terpinene	0.50	0.50	*1.00*	1.00	1.33	2.33	0.89	1.33	2.22
	±Linalool	0.50	0.73	1.23	0.50	0.50	*1.00*	0.44	0.67	1.11
	Eugenol	0.25	0.60	*0.82*	0.25	0.50	*0.75*	0.22	0.67	*0.89*
	Carvacrol	0.25	0.50	*0.75*	0.25	0.67	*0.92*	0.11	0.50	*0.61*
	Cinnamaldehyde	0.13	1.20	1.33	0.13	0.50	*0.63*	0.05	0.60	*0.65*

^1^ FIC(A)—fractional inhibitory concentration of antibiotic; ^2^ FIC(B)—fractional inhibitory concentration of EOC; ^3^ ΣFIC—sum of FIC values; **bold** values indicate synergy, while italicized values indicate additivity.

**Table 3:** The FIC values of combinations of conventional antibiotics (A) and EOCs (B) against Gram-negative bacteria (*n* = 4).

Conventional Antibiotic	Essential Oil Compounds	*P. aeruginosa* (ATCC 27853)			*A. baumannii* (ATCC 19606)		
		FIC(A) ^1^	FIC(B) ^2^	ΣFIC ^3^	FIC(A)	FIC(B)	ΣFIC
**Ciprofloxacin**	α-pinene	0.54	0.27	*0.81*	1.20	0.50	1.70
	γ-terpinene	0.54	0.29	*0.83*	1.20	0.50	1.70
	±Linalool	0.54	0.33	*0.87*	1.20	1.00	2.20
	Eugenol	0.27	0.33	*0.60*	0.60	0.67	1.27
	Carvacrol	0.27	0.67	*0.94*	0.60	1.00	1.60
	Cinnamaldehyde	0.07	0.25	**0.32**	0.30	1.00	1.30
**Gentamicin**	α-pinene	0.75	0.10	*0.85*	0.57	0.25	*0.82*
	γ-terpinene	1.51	0.21	1.72	1.14	0.50	1.64
	±Linalool	2.00	0.17	2.17	0.57	0.50	1.07
	Eugenol	1.00	0.33	1.33	0.29	0.33	*0.62*
	Carvacrol	0.50	0.33	*0.83*	0.57	1.00	1.57
	Cinnamaldehyde	1.00	1.00	2.00	0.29	1.00	1.29
**Meropenem**	α-pinene	2.00	0.53	2.53	1.04	0.25	1.29
	γ-terpinene	1.00	0.13	1.13	1.04	0.25	1.29
	±Linalool	1.00	0.17	1.17	1.04	0.50	1.54
	Eugenol	0.75	0.25	*1.00*	0.39	0.25	*0.64*
	Carvacrol	0.75	0.50	1.25	0.39	0.38	*0.77*
	Cinnamaldehyde	0.50	0.50	*1.00*	0.26	0.50	*0.76*
**Tetracycline**	α-pinene	0.33	0.53	*0.86*	0.40	0.25	*0.65*
	γ-terpinene	0.33	0.57	*0.90*	0.60	0.38	*0.98*
	±Linalool	0.33	0.67	*1.00*	0.80	1.00	1.80
	Eugenol	0.33	1.33	1.66	0.40	0.67	1.07
	Carvacrol	0.17	1.33	1.50	0.40	1.00	1.40
	Cinnamaldehyde	0.08	1.00	1.08	0.20	1.00	1.20

^1^ FIC(A)—fractional inhibitory concentration of antibiotic; ^2^ FIC(B)—fractional inhibitory concentration of EOC; ^3^ ΣFIC—sum of FIC values; **bold** values indicate synergy, while italicized values indicate additivity.

**Table 4:** The FIC values of combinations of antifungals (A) and EOCs (B) against *C. albicans* (*n* = 4).

Antifungals	Essential Oil Compounds	*C. albicans* (ATCC 10231)		
		FIC(A) ^1^	FIC(B) ^2^	ΣFIC ^3^
**Miconazole**	α-pinene	0.80	1.33	2.13
	γ-terpinene	0.80	0.80	1.60
	±Linalool	0.40	0.80	1.20
	Eugenol	0.20	0.67	*0.87*
	Carvacrol	0.15	0.75	*0.90*
	Cinnamaldehyde	0.05	1.00	1.05
**Nystatin**	α-pinene	0.40	1.33	1.73
	γ-terpinene	0.40	0.80	1.20
	±Linalool	0.20	1.33	1.53
	Eugenol	0.10	0.67	*0.77*
	Carvacrol	0.10	1.00	1.10
	Cinnamaldehyde	0.03	1.00	1.03

^1^ FIC(A)—fractional inhibitory concentration of antibiotic; ^2^ FIC(B)—fractional inhibitory concentration of EOC; ^3^ ΣFIC—sum of FIC values; italicized values indicate additivity.

**Table 5:** The mean percentage mortality and standard deviation (±SD) for the conventional antibiotics and controls (*n* = 3).

Conventional Antibiotics	Concentrations			
	0.01 mg/mL		0.05 mg/mL	
	24 h	48 h	24 h	48 h
**Amoxicillin**	0.00 ± 0.00	2.67 ± 3.06	0.00 ± 0.00	0.67 ± 1.15
**Ciprofloxacin**	0.00 ± 0.00	0.00 ± 0.00	1.67 ± 2.89	3.67 ± 6.35
**Erythromycin**	1.00 ± 2.31	5.67 ± 1.15	11.00 ± 7.94	15.67 ± 7.77
**Gentamicin**	0.00 ± 0.00	0.00 ± 0.00	2.67 ± 2.31	7.00 ± 2.64
**Meropenem**	0.00 ± 0.00	0.00 ± 0.00	0.00 ± 0.00	0.00 ± 0.00
**Tetracycline**	1.00 ± 1.15	6.00 ± 5.29	5.67 ± 4.73	12.00 ± 3.61
**Miconazole**	0.00 ± 0.00	0.00 ± 0.00	7.67 ± 3.06	17.00 ± 1.73
**Nystatin**	3.00 ± 2.31	7.67 ± 3.79	5.33 ± 7.57	12.00 ± 7.94
**Controls**		**24 h**	**48 h**	
Potassium dichromate (positive control)		100.00 ± 0.00	100.00 ± 0.00	
2.00% DMSO (negative control)		3.00 ± 1.41	4.50 ± 0.71	
Distilled water (negative control)		0.00 ± 0.00	0.00 ± 0.00	
Saltwater (negative control)		1.93 ± 0.03	2.10 ± 0.01	

**Table 6:** Lethal concentration (LC_50_) with a 95% confidence interval (CI) of EOCs after 24 h and 48 h.

Essential Oil Compounds	LC_50_ (µg/mL) at 24 h	LC_50_ (µg/mL) at 48 h
**α-pinene**	>1000 ^1^ (>1000)	>1000 (>1000)
**γ-terpinene**	>1000 (>1000)	>1000 (>1000)
**±Linalool**	73.04 (71.24–74.85)	84.12 (82.23–86.01)
**Eugenol**	77.62 (75.41–79.83)	56.47 (54.80–58.14)
**Carvacrol**	64.43 (62.46–66.40)	37.14 (35.67–38.61)
**Cinnamaldehyde**	74.01 (71.91–76.11)	64.05 (62.26–65.84)

^1^ The LC_50_ value represents the concentration of a test substance necessary to have a lethal effect on 50% of a brine shrimp sample. LC_50_ values < 249 µg/mL are considered highly toxic; 250–499 µg/mL are considered moderately toxic; 500–999 µg/mL are considered weak or low in toxicity; and ≥1000 µg/mL are considered non-toxic [84].

**Table 7:** Lethal concentration (LC_50_) with a 95% confidence interval (CI) of synergistic combinations after 24 h and 48 h.

Antimicrobial Synergistic Combination	LC_50_ (µg/mL) at 24 h	LC_50_ (µg/mL) at 48 h
**Amoxicillin + Carvacrol**	522.95 (520.99–524.91)	513.89 (511.77–516.01)
**Gentamicin + Carvacrol**	764.77 (762.61–766.94)	696.07 (693.85–698.29)
**Miconazole + Carvacrol**	707.03 (704.83–709.24)	657.45 (655.12–659.78)
**Ciprofloxacin + Cinnamaldehyde**	827.92 (825.85–829.99)	640.04 (638.25–641.83)
**Erythromycin + Cinnamaldehyde**	736.85 (734.84–738.86)	573.63 (571.86–575.40)
**Miconazole + Cinnamaldehyde**	704.23 (702.34–706.12)	636.05 (633.99–638.11)
**Amoxicillin + Eugenol**	806.43 (804.42–808.44)	628.81 (626.77–630.85)

**Table 8:** Lethal concentration (LC_50_) and selectivity index (SI) of synergistic ratios after 24 h and 48 h.

Synergistic Combinations	Pathogen	Synergistic Ratios (AB:EOC)	LC_50_ (µg/mL) of Combinations at Synergistic Ratios		SI of Combinations at Synergistic Ratios	
			24 h	48 h	24 h	48 h
**Amoxicillin + ** **Carvacrol**	*S. aureus*	60:40	*612.03* ^1^	*601.54*	1.53	1.50
		50:50	*522.95*	*513.89*	1.05	1.03
		40:60	433.87	426.24	0.87	0.85
		30:70	344.79	338.60	0.98	0.97
		20:80	255.71	250.95	0.32	0.31
**Gentamicin + ** **Carvacrol**	*S. aureus*	70:30	**1025.32** ^2^	*931.44*	3.42	3.10
		60:40	*895.04*	*813.75*	***4.47*** ^3^	***4.07***
		50:50	*764.77*	*696.07*	3.06	2.78
		40:60	*634.49*	*578.38*	2.11	1.93
		30:70	*504.21*	460.69	0.72	0.66
**Miconazole + ** **Carvacrol**	*S. aureus*	60:40	*826.80*	*769.44*	2.07	1.92
		50:50	*707.03*	*657.45*	2.83	2.63
		40:60	*587.25*	*545.45*	1.96	1.82
		30:70	467.48	433.46	1.34	1.24
**Miconazole + ** **Cinnamaldehyde**	*S. aureus*, *S. epidermidis*	70:30	*944.15*	*852.75*	3.14	2.84
		60:40	*824.19*	*744.40*	***4.12***	3.72
		50:50	*704.23*	*636.05*	***5.63***	***5.09***
		40:60	*584.27*	*527.70*	3.89	3.52
		30:70	464.31	419.35	1.32	1.20
**Ciprofloxacin + ** **Cinnamaldehyde**	*P. aeruginosa*	60:40	*968.95*	*749.06*	***9.68***	***7.49***
		50:50	*827.92*	*640.04*	***6.62***	***5.12***
		40:60	*686.88*	*531.01*	***4.58***	3.54
		30:70	*545.85*	421.98	3.12	2.41
		20:80	404.81	312.95	1.01	0.78
**Erythromycin + ** **Cinnamaldehyde**	*S. epidermidis*	70:30	987.89	769.06	6.58	5.12
		60:40	*862.37*	*671.34*	***4.31***	3.36
		50:50	*736.85*	*573.63*	***5.89***	***4.59***
		40:60	*611.33*	475.92	***4.07***	3.17
		30:70	485.81	378.20	1.39	1.08
**Amoxicillin + ** **Eugenol**	*C. acnes*	70:30	**1081.17**	843.04	***14.41***	***11.23***
		60:40	*943.80*	*735.93*	***9.43***	***7.36***
		50:50	*806.43*	*628.81*	***6.45***	***5.03***
		40:60	*669.05*	*521.69*	***4.46***	3.48
		30:70	*531.68*	414.58	3.04	2.37

^1^ Italicized values indicate a weak or low toxicity; ^2^ bold values indicate non-toxic concentrations; and ^3^ bold and italicized values indicate SI ≥ 4.

## Data Availability

Data are contained within the article and Supplementary Materials.

## Supplementary Materials

The following supporting information can be downloaded at https://www.mdpi.com/article/10.3390/molecules29061225/s1.

## Abbreviations

EO	Essential oil
EOC	Essential oil compound
FIC	Fractional inhibitory concentration
LC	Lethal concentration
SI	Selectivity index
