# Supplementary material for: Essential Oil Compounds in Combination with Conventional Antibiotics for Dermatology
Source: Molecules. 2024 Mar 8;29(6):1225. doi: 10.3390/molecules29061225 (PMC10974782; doi:10.3390/molecules29061225)

Essential oil compounds in combination with conventional antibiotics for dermatology

S. Simbu[1], A. Orchard[1], S. van Vuuren[1]

[1] Department of Pharmacy and Pharmacology, Faculty of Health Sciences, University of the Witwatersrand, Parktown, South Africa, 2193

Supplementary Data

Table S1. Synergistic interactions against *S. aureus*.

Isobologram plot number	Ratio volumes (µl) (Antibiotic: EOC)	Amoxicillin + Carvacrol		Gentamicin + Carvacrol		Miconazole + Carvacrol		Miconazole + Cinnamaldehyde	
		MIC of antibiotic in combination (µg/mL)	MIC of EOC in combination (µg/mL)	MIC of antibiotic in combination (µg/mL)	MIC of EOC in combination (µg/mL)	MIC of antibiotic in combination (µg/mL)	MIC of EOC in combination (µg/mL)	MIC of antibiotic in combination (µg/mL)	MIC of EOC in combination (µg/mL)
**1**	90:10	1.13	400	1.13	400	1.13	400	1.13	400
**2**	80:20	0.50	400	0.50	400	0.50	400	0.50	400
**3**	70:30	0.44	600	0.22	300	0.22	300	0.22	300
**4**	60:40	0.19	400	0.09	200	0.19	400	0.09	200
**5**	50:50	0.16	500	0.08	250	0.08	250	0.04	125
**6**	40:60	0.06	500	0.06	300	0.06	300	0.03	150
**7**	30:70	0.05	350	0.09	700	0.05	350	0.05	350
**8**	20:80	0.06	800	0.13	1600	0.06	800	0.03	400
**9**	10:90	0.06	1800	0.06	1800	0.03	900	0.02	450

Table S2. Synergistic interactions against *S. epidermidis*, *C. acnes* and *P. aeruginosa.*

Isobologram plot number	Ratio volumes (µl) (Antibiotic: EOC)	***S. epidermidis*** **(ATCC 12228)**				***C. acnes*** **(ATCC 11827)**		***P. aeruginosa*** **(ATCC 27853)**	
		**Erythromycin + Cinnamaldehyde**		**Miconazole + Cinnamaldehyde**		**Amoxicillin + Eugenol**		**Ciprofloxacin + Cinnamaldehyde**	
		**MIC of antibiotic in combination (µg/mL)**	**MIC of EOC in combination (µg/mL)**	**MIC of antibiotic in combination (µg/mL)**	**MIC of EOC in combination (µg/mL)**	**MIC of antibiotic in combination (µg/mL)**	**MIC of EOC in combination (µg/mL)**	**MIC of antibiotic in combination (µg/mL)**	**MIC of EOC in combination (µg/mL)**
**1**	90:10	0.28	100	1.13	400	0.14	50	0.29	100
**2**	80:20	0.25	200	0.50	400	0.13	100	0.13	100
**3**	70:30	0.11	150	0.22	300	0.05	75	0.11	150
**4**	60:40	0.09	200	0.09	200	0.05	100	0.05	100
**5**	50:50	0.04	125	0.04	125	0.04	125	0.04	125
**6**	40:60	0.03	150	0.03	150	0.03	150	0.03	150
**7**	30:70	0.05	350	0.05	350	0.02	175	0.02	175
**8**	20:80	0.06	800	0.06	800	0.03	400	0.03	400
**9**	10:90	0.06	1800	0.03	900	0.03	900	0.03	900

Figure S1. Study design.

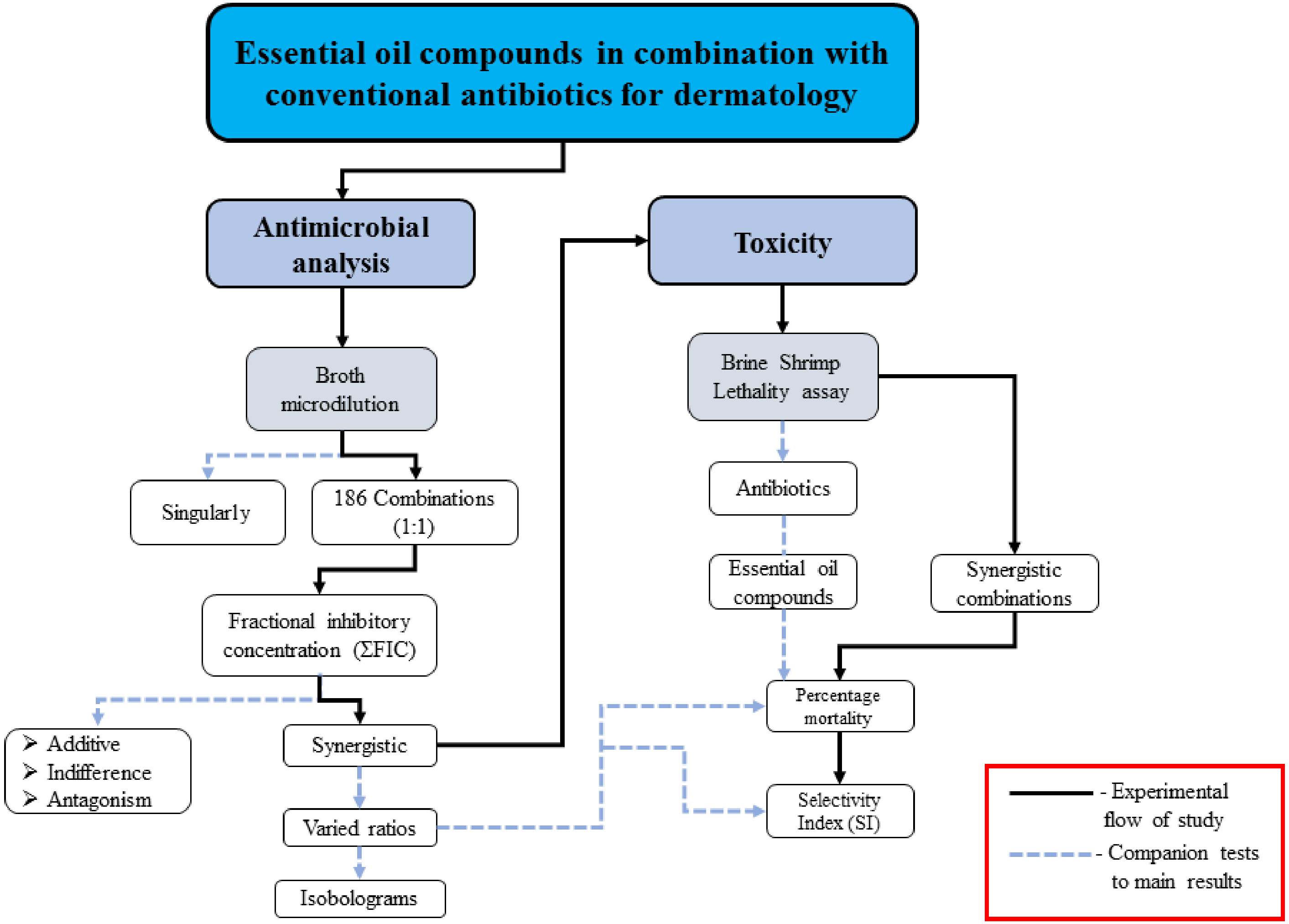

Supplement: Supplementary file 1 [file molecules-29-01225-s001.zip › molecules-2883905-supplementary.pdf]
